# Quint: An R package for the identification of subgroups of clients who differ in which treatment alternative is best for them

**DOI:** 10.3758/s13428-015-0594-z

**Published:** 2015-06-20

**Authors:** Elise Dusseldorp, Lisa Doove, Iven van Mechelen

**Affiliations:** Department of Psychology, Katholieke Universiteit Leuven, Leuven, Belgium; Mathematical Institute, Leiden University, PO Box 9512, 2300 RA Leiden, The Netherlands; Netherlands Organization for Applied Scientific Research (TNO), Leiden, The Netherlands

**Keywords:** Treatment-subgroup interaction, Moderator, Treatment efficacy, Subgroup analysis, Regression trees, Computer software

## Abstract

In the analysis of randomized controlled trials (RCTs), treatment effect heterogeneity often occurs, implying differences across (subgroups of) clients in treatment efficacy. This phenomenon is typically referred to as treatment-subgroup interactions. The identification of subgroups of clients, defined in terms of pretreatment characteristics that are involved in a treatment-subgroup interaction, is a methodologically challenging task, especially when many characteristics are available that may interact with treatment and when no comprehensive a priori hypotheses on relevant subgroups are available. A special type of treatment-subgroup interaction occurs if the ranking of treatment alternatives in terms of efficacy differs across subgroups of clients (e.g., for one subgroup treatment A is better than B and for another subgroup treatment B is better than A). These are called qualitative treatment-subgroup interactions and are most important for optimal treatment assignment. The method QUINT (Qualitative INteraction Trees) was recently proposed to induce subgroups involved in such interactions from RCT data. The result of an analysis with QUINT is a binary tree from which treatment assignment criteria can be derived. The implementation of this method, the R package quint, is the topic of this paper. The analysis process is described step-by-step using data from the Breast Cancer Recovery Project, showing the reader all functions included in the package. The output is explained and given a substantive interpretation. Furthermore, an overview is given of the tuning parameters involved in the analysis, along with possible motivational concerns associated with choice alternatives that are available to the user.

## Introduction

In the field of evidence-based health and mental health care, the gold standard method to establish treatment efficacy is a randomized controlled trial (RCT). In such a trial, clients with a certain problem or disorder are randomly assigned to one out of (at least) two treatments (e.g., two alternative treatments, or one alternative treatment versus treatment as usual). In the analysis of the resulting data, for a long time the usual research question has been: Which treatment is, on average, most effective? This question typically reflects the goal of determining the best treatment for all clients with the problem or disorder under study within a one-size-fits-all approach. The merit of such an approach is obvious: Every client with the problem in question then can be assigned to the best treatment, regardless of her or his individual characteristics (Fierz 2004).

When the difference in efficacy between the treatments under study is not equal across all subgroups of clients, that is, differential treatment efficacy is present, this reflects statistically speaking a treatment-subgroup interaction (or treatment-covariate interaction). A special type of such an interaction occurs if for some subgroups of clients treatment A is more effective than B, while for other subgroups the reverse holds true. This is called a disordinal (Lubin 1961) or qualitative (Byar 1985) treatment-subgroup interaction. By contrast, if in all subgroups the same treatment is more effective than the other, and the subgroups only differ in the magnitude of the treatment effect, the treatment-subgroup interaction is called ordinal or quantitative. For optimal treatment assignment in clinical practice, quantitative interactions are less consequential, because optimal assignment would simply come down to assigning all clients to the same treatment (i.e., the marginally best treatment alternative). However, qualitative interactions imply that some subgroups of clients should be treated differently than other subgroups, and are therefore most relevant for clinical practice (Byar 1985). For example, in case of two treatments A and B, qualitative interactions imply that some subgroups should preferably be assigned to A whereas other subgroups should preferably be assigned to B. From the client characteristics that define the subgroups involved in a qualitative treatment-subgroup interaction, rules for an optimal treatment assignment can be derived. In this way, therapies tailored to the client can be realized, which are of key interest in the field of personalized health care (Fierz 2004). Over the last few decades, this field has become increasingly important. By focusing on the question: What works for whom? personalized health care can be regarded as a movement away from the one-size-fits-all approach (Roth and Fonagy 2006).

When a priori hypotheses are available about possible moderator variables or when the number of pretreatment characteristics is small, several statistical methods exist to examine treatment-subgroup interactions. Examples include analysis of variance with prespecified contrast codings (Shaffer 1991), and moderated regression analysis (Cohen, Cohen, West, & Aiken, 2003). (For a description of moderated regression analysis within the framework of randomized controlled trials, see Kraemer, Wilson, Fairbun, & Agras, 2002). In practice, however, it often occurs that no clear or comprehensive a priori hypotheses on relevant subgroups are available, and that many pretreatment characteristics have been measured. In such exploratory situations, it is quite a task to identify the characteristics of subgroups that are involved in treatment-subgroup interactions and, at the same time, to control for inferential errors involved in hypothesis testing (i.e., type I and type II errors).

Over the last decade, a group of tree-based methods has been developed to deal with this type of exploratory situations, including STIMA (simultaneous threshold interaction modeling; Dusseldorp, Conversano, & Van Os, 2010; Dusseldorp & Meulman 2004), Interaction Trees (Su, Tsai, Wang, Nickerson, & Li, 2009), Model-based recursive partitioning (Zeileis, Hothorn, & Hornik, 2008), Virtual Twins (Foster, Taylor, & Ruberg, 2011), and SIDES (subgroup identification based on differential effect search; Lipkovich, Dmitrienko, Denne, & Enas, 2011). All these methods rely on a recursive partitioning type of algorithm with cross-validation or bootstrap-based bias-correction procedures to control for inferential errors. A major difference between these methods is that Virtual Twins and SIDES focus on the extraction of only those subgroups in which the effect of an alternative treatment is considerably larger compared to a reference treatment, whereas the solutions found by the other three methods represent the full group of persons (for a more extensive comparison, see Doove, Dusseldorp, Van Deun, & Van Mechelen, 2014). Yet, a shortcoming of the methods in question is that the user is not given any control over the type of interactions (qualitative or quantitative) resulting from the analysis. This is regrettable because, as was mentioned before, especially qualitative treatment-subgroup interactions have serious consequences for optimal (personalized) treatment assignment. Another shortcoming of these methods is that the accompanying software lacks instructions about how to use it to identify treatment-subgroup interactions. Some of these methods merely provide software code without a manual (e.g., SIDES) and some of them provide only general instructions that are not adapted to treatment-subgroup interactions (e.g., STIMA).

As a solution, recently a new tree-based method, called Qualitative INteraction Trees (QUINT), was proposed (Dusseldorp and Van Mechelen 2014). QUINT was specifically designed to induce subgroups that are involved in *qualitative* treatment-subgroup interactions. In the present paper, we introduce the corresponding software quint, which is a package in R, a free software environment (R Core Team 2014). The R package can be installed from the CRAN repository. Our aims are to explain and illustrate the usage of the package to study qualitative treatment-subgroup interactions in behavioral research. The following issues will be discussed: For which type of data is the package suitable, which steps need to be taken during the analysis process, what are the available choice options for the analysis, and how to postprocess and interpret the output of quint. The reader is guided through the analysis process by means of data from the Breast Cancer Recovery Project (Scheier et al. 2007). Below, we start with a short conceptual outline of the method. Subsequently, the software will be introduced.

## The method QUINT

### Goal of QUINT

**Fig. 1:**
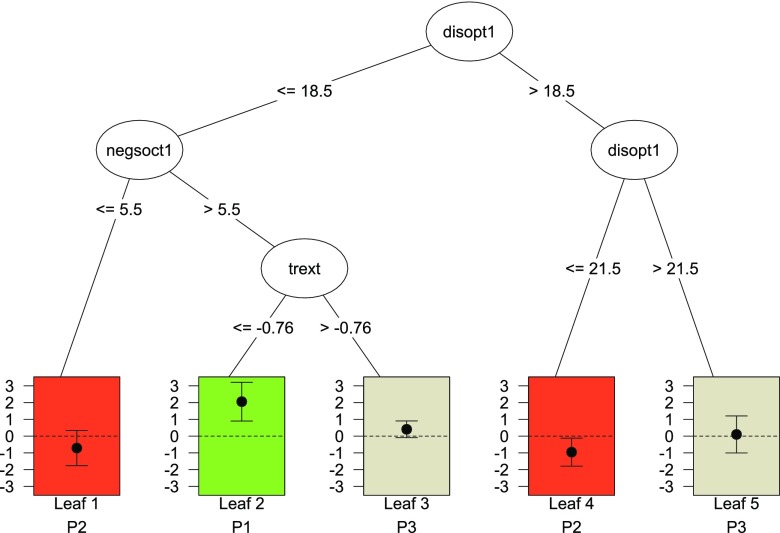
Example of a pruned qualitative interaction tree for the outcome Improvement in depression using the Breast Cancer Recovery Project data, as produced by the package quint. The splitting variables are: disopt (dispositional optimism), negsoct1 (negative social interaction), and trext (treatment extensiveness index). Each leaf of the tree is assigned to one of the three subgroups *℘*_1_, *℘*_2_, or *℘*_3_, denoted in the figure by P1, P2, and P3, respectively, and visualized by different colors of the leaves (*green*, *red*, and *grey*). The *vertical axis* of the leaves pertains to the effect size *d*

QUINT is a tree-based clustering method for data obtained from a two-arm RCT that include an outcome variable and a number of variables measured before treatment started (i.e., at baseline). These baseline variables are typically client characteristics, but could also be therapist characteristics or characteristics of the setting and so on. The aim of QUINT is to identify three subgroups of clients (i.e., partition classes), each of which comprises one or more client types as defined by different combinations of client characteristics. Subgroup *℘*_1_ contains those clients for whom Treatment A is better than Treatment B, Subgroup *℘*_2_ those for whom B is better than A, and (the optional) Subgroup *℘*_3_ those for whom it does not make any difference. In the latter group, the difference in treatment outcome between A and B is negligible (also called by others “the region of uncertainty”, Shuster & van Eys, 1983). The subgroups and client types are not known beforehand, but are to be induced during the data analysis. Therefore, QUINT can be considered an unsupervised learning method (Hastie et al. 2001). The subgroups are represented by a binary tree. For an example of such a tree, one may refer to Fig. 1, which is a representation of a tree produced by the package quint. The root node (i.e., the ellipse at the top) represents the total group of clients. Each split of the tree divides a node into two child nodes on the basis of a threshold value (i.e., a split point) on a client characteristic. For example, the root node in Fig. 1 is split into two internal nodes on the basis of the value 18.5 on the variable “disopt1”. Clients who score 18.5 or lower fall into the left child node and the others fall into the right child node. How the splitting variable and split point are chosen will be explained below (see “the QUINT algorithm”). Each leaf or end node of the tree (in Fig. 1 the leaves are displayed by rectangles) represents a client type and is assigned to one of the three subgroups, colored in green, red, or grey. A green leaf belongs to Subgroup *℘*_1_, a red leaf to Subgroup *℘*_2_, and a grey leaf to Subgroup *℘*_3_. Note that several leaves can be assigned to the same subgroup, for example, both Leaf 1 and Leaf 4 in Fig. 1 are assigned to *℘*_2_.

The underlying goal of QUINT is to identify subgroups that are involved in an *optimal* qualitative treatment-subgroup interaction. Optimality comprises two components here: (a) In both Subgroup *℘*_1_ and Subgroup *℘*_2_, the absolute difference in treatment outcome between A and B should be as high as possible, and (b) the sample sizes of both Subgroup *℘*_1_ and *℘*_2_ should be as large as possible (to avoid trivial interactions based on small subgroups of clients only). The advantage of also having the possibility to assign patients to a Subgroup *℘*_3_ is that the difference in treatment outcome in Subgroups *℘*_1_ and *℘*_2_ can be increased. The two optimality components are referred to, respectively, as the Difference in treatment outcome component and the Cardinality component. Both components are taken into account by the partitioning criterion (*C*) of QUINT (for formula, see Dusseldorp & Van Mechelen, 2014). With regard to the Difference in treatment outcome component, two options are available: (1) the standardized mean difference, expressed as Cohen’s effect size *d* (Cohen 1988), and (2) the raw mean difference in treatment outcome between the groups. The resulting two possible specifications of the partitioning criterion are referred to as the Effect size criterion and the Difference in means criterion, respectively. Which criterion is most appropriate depends on the research problem at hand, as will be discussed in the section on the R package quint.

### The QUINT algorithm

The QUINT partitioning criterion *C* is maximized using a sequential partitioning algorithm, that is, a stepwise binary splitting procedure. In the Appendix, a flowchart of the algorithm is shown, together with a description. The algorithm starts with all clients in the root node; this node is split into two child nodes on the basis of a threshold value on one baseline characteristic. Subsequently, one of these two child nodes is split, etc. (see e.g., Fig. 1). In each step of the splitting procedure, the node, the baseline characteristic, the admissible split point for that characteristic, and the admissible assignment of the leaves of the resulting tree to the three subgroups (*℘*_1_, *℘*_2_, or *℘*_3_) are chosen that maximize the QUINT criterion *C*. Note that a split point and an assignment are considered admissible if they satisfy a number of boundary conditions (see further below). Also, note that the specific assignment of the leaves to *℘*_1_, *℘*_2_, or *℘*_3_ depends on the value of criterion *C*, and not on a significance test of the difference in treatment outcome. For example, a node can be assigned to *℘*_2_, while the 95 *%* confidence interval of the effect size includes a 0 (e.g., Leaf 1 in Fig. 1). If the new maximum value of *C* is higher than the current value, a split is performed, and the algorithm proceeds to the next step, in which the whole procedure is repeated. The repetition of the *whole* procedure implies that after each split of the tree, all leaves of the tree are re-assigned to the subgroups *℘*_1_, *℘*_2_, or *℘*_3_.

The algorithm stops automatically if no candidate parent node can be found with an admissible triplet (i.e., baseline characteristic, split point, and assignment to subgroup) and a higher value of *C* than the current value, or if the current total number of leaves equals a user-specified maximum. The boundary conditions to determine the admissibility of a split are:

1. *The qualitative interaction condition*: After the first split, in each of the two resulting leaves the absolute value of the treatment effect size, that is, Cohen’s *d* should exceed a critical minimum value (*d*_*min*_). To ensure that this condition is independent of the type of outcome measure, the standardized effect size is used here (rather than the raw difference in means). As mentioned before, the QUINT analysis immediately stops (i.e., no tree is fitted) if this condition is not satisfied.
2. *The minimal sample size per treatment condition*: A minimum number of clients assigned to treatment A and B is needed in each leaf of the tree.
3. *The nonempty partition class condition*: Partition classes *℘*_1_ and *℘*_2_ should be nonempty. Partition class *℘*_3_ may be empty.
4. *The mean difference per node condition*: A leaf can only be assigned to *℘*_1_ if the mean outcome of the clients in treatment A is higher than the mean outcome of those in treatment B; conversely, a node can only be assigned to *℘*_2_ if the mean outcome of treatment B is higher than the mean outcome of treatment A.

The first condition concerns the admissibility of the first split. It is a check whether a qualitative interaction is present in the data, and helps to control for the type I error rate (i.e., the risk of identifying spurious qualitative interactions). The second condition pertains to the admissibility of a split point, and the final two to the admissibility of a leaf assignment. The first two boundary conditions include tuning parameters that can be specified by the user (see section R package quint).

With every split of the tree, the fit of the tree increases and the tree may become very large and complex. The resulting final tree may model the noise in the data in addition to the true signals. As a result, the final tree may satisfactorily fit the training data, but may not fit future data. To overcome this so-called overfitting (Hastie et al. 2001), the tree needs to be pruned back.

For this pruning, we start from the observation that the QUINT algorithm results in a series of nested subtrees of sizes varying from two leaves to the number of leaves when the algorithm stopped. Each of these subtrees goes with an apparent value of the QUINT partitioning criterion, which is usually biased because the subtree fits the data too well. In other words, the apparent fit (i.e., the “observed fit”) is overoptimistic, because it is estimated using the total sample (i.e., the original data) as training data, and based on a greedy search of each variable and each possible split point. Therefore, the criterion values are subjected to a bootstrap-based bias correction procedure (LeBlanc and Crowley 1993) making use of QUINT analyses of *B* bootstrap samples drawn from the original data. This procedure implies that for each subtree of size *L*, the amount of optimism is estimated in the following way: Each bootstrap sample is subjected to a QUINT analysis, which results in a series of nested bootstrap subtrees. We then select from this series, the bootstrap subtree of size *L*. The value of the partitioning criterion for this bootstrap subtree is computed (i.e., training value). Then, the bootstrap subtree is “frozen” (i.e., splitting variables, split points, and assignments to the classes are fixed) and applied to the original data (which are now used as test data) and the test value of the partitioning criterion is computed. The amount of optimism is then calculated as the difference between the training value and the test value and by subsequently averaging this across all bootstrap samples (see Appendix B in the Supplementary materials of Dusseldorp & Van Mechelen, 2014). Subsequently, this amount can be subtracted from the apparent criterion value for the subtree of size *L* as obtained from the QUINT analysis of the original data, resulting in a bias-corrected criterion value for that subtree. Finally, the optimal pruned tree size is selected using a so-called one standard error rule (Breiman, Friedman, Olshen, & Stone, 1984), meaning that the most parsimonious subtree is chosen whose bias-corrected criterion value is no more than one standard error below the maximum bias-corrected criterion value (with the standard error being derived from the standard deviation of the optimism across the bootstrap samples of each tree size under study).

## Motivating example

As a guiding example, we will use data from the Breast Cancer Recovery Project (BCRP) for younger women with early stage breast cancer who previously underwent a lumpectomy and received combined radiation and chemotherapy (Scheier et al. 2007). The participating women in this clinical trial were randomly assigned to one of three therapy conditions: a nutrition intervention (*n*=85), an education intervention (*n*=83), and a control condition (*n*=84). The women were measured at three time points: at baseline, at 4 months (i.e., immediately after treatment) and at 13 months (i.e., 9 months post-treatment). The primary outcome variables were measures of depression (CES-D; Radloff, 1977) and health-related quality-of-life (i.e., physical and mental functioning; two subscales of the SF-36; Ware & Sherbourne, 1992). In a first paper on the BCRP (Scheier et al. 2005), it was shown that both the nutrition intervention and the education intervention were superior compared to the control. In a second paper (Scheier et al. 2007), it was investigated whether the main effects of the two interventions were moderated by one of the following baseline characteristics:

- demographic variables: age, gender, and nationality;
- indicators of treatment severity: weight change, treatment extensiveness index (created by standardizing and aggregating type of surgery [lumpectomy or mastectomy] with type of adjuvant treatment received [none, radiation or chemotherapy, both]), and comorbidity (sum of the checked potential comorbidities, such as, diabetes, migraines, arthritis, or angina, and the reported conditions that the participant currently had [open question]);
- personality characteristics: dispositional optimism (a global expectation that more good and desirable things will happen than their bad and undesirable counterparts; Scheier, Carver, & Bridges, 1994), unmitigated communion (a focus on others to the exclusion of the self; Fritz & Helgeson, 1998), and negative social interaction.

In our reanalysis of these data, we will focus on the comparison of the nutrition and education intervention making use of the above-mentioned client characteristics. The same outcome variables are used as in the study of Scheier et al. 2007: sum scores of a depression scale, and a physical functioning scale, both measured at baseline and at the 9- month post-treatment follow-up. More specifically, the change scores from baseline to follow-up are used in the analysis. For physical functioning, guidelines for clinically important changes were available in the literature: change scores of 5, 20, and 30, can be judged as, respectively, a small, medium, and large difference (Wyrwich et al. 2005). Our central research question is: For which subgroup of women is a nutrition intervention more effective than an education intervention, for which subgroup does the reverse hold true, and for which subgroup do the two interventions not lead to clearly different outcomes?

## R package: quint

### Preparation

Install the latest version of the R software environment (R Core Team 2014). In the menu of R, go to Packages, install the package quint from CRAN, and load the package. 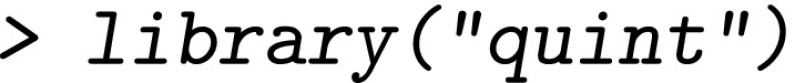


### Input: Data and formula

The study design for the data to be analyzed with quint needs to be a randomized controlled trial. The data structure in R can be an R data frame or an R matrix. The data set has to include at least the following variables, the order and names of which are not important: one continuous outcome variable (with the class of this variable being numeric), a dichotomous treatment variable (i.e., class may be factor or numeric), and several baseline characteristics (i.e., candidate splitting variables) that can be ordinal or continuous (i.e., class is numeric), or dichotomous (i.e., categorical variables with only two categories, such as gender or continuous characteristics that are dichotomized using a prespecified clinically informed cut-off score). The current version of quint is restricted to a dichotomous treatment variable and can handle neither categorical baseline characteristics with more than two categories, nor categorical outcome variables. Our example data set is included in the R package and can be inspected in the following way: 
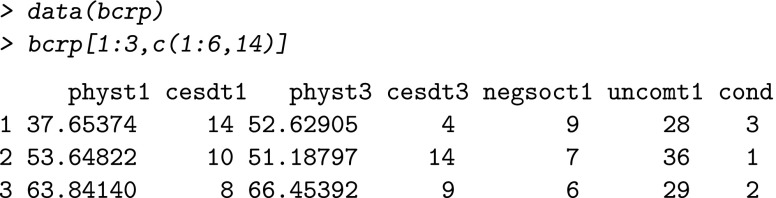


The two outcome variables, physical functioning (phys) and depression (cesd), have been measured at baseline (t1) and at 9 months post-treatment (t3). The variables negative social interaction (negsoct1) and unmitigated communion (uncomt1) are patient characteristics measured at baseline (i.e., a selection of the nine characteristics in this data set). The treatment variable cond represents three therapy conditions (nutrition, education, and control condition, denoted by 1 to 3, respectively). To get more insight into the meaning of the variables, one may use the help function: 
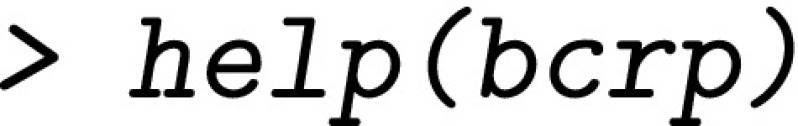


If a data set contains more than two treatment conditions, as in this data set, the user needs to select two conditions of interest, before performing a quint analysis. As we focus in this example on the comparison between the nutrition and the education condition, we create a new data set without the third condition by the following command: 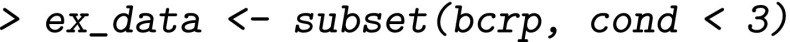


Before the analysis, the user needs to specify the role of all variables by means of a formula, which looks as follows: *Y*~*T*|*X*_1_+...+*X*_*J*_, with a single outcome variable *Y* followed by two parts separated by the symbol |. The first part represents the dichotomous treatment variable *T* and the second part the baseline characteristics *X*_1_ to *X*_*J*_, where *J* equals the total number of baseline characteristics under study. The order of *X*_1_ to *X*_*J*_ within the second part of the formula is arbitrary. In general, the outcome *Y* may be a single follow-up measure, a change or rate of change score from baseline to follow-up, a follow-up score adjusted for baseline, or a variable indicating time to an event. (Note that if outcome measurements at more than two time points would be available, quint analyses could be run on change scores between any two time points of interest.) We recommend to use outcome variables measured on scales that are calibrated in terms of what constitutes clinically meaningful differences. Furthermore, we recommend to construct the outcome variable in such a way that a higher score indicates a better treatment outcome to facilitate the interpretation of the output.

For our example data, we create two formulas, one for each outcome variable. For change in depression, the formula is specified as follows: 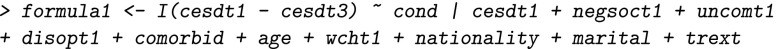


In the above formula, the expression I (cesdt1 - cesdt3) is used to calculate the change score. The posttest depression score (cesdt3) is subtracted from the baseline (cesdt1) to ensure that a higher score indicates a better treatment outcome, that is, a larger improvement in depression. Furthermore, the nine patient characteristics are listed as candidate splitting variables, in addition to the baseline measurement of the outcome variable.

For change in physical functioning, the formula is specified as follows: 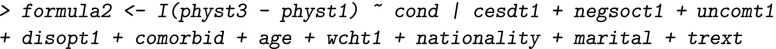


In the above formula, the baseline score is subtracted from the posttest score to ensure that a higher score indicates a larger improvement in physical functioning.

### First analyses with default values of parameters

We now start with the first analyses using the main function (called quint) of the package with default values for the tuning parameters. In the next section, an overview of the tuning parameters is given, and it will be shown how (and why) default values can be changed. Just before running the code, we fix the seed to be able to replicate the results of the bootstrapping. During the analysis, screen output is generated automatically to enable the user to follow the process. 
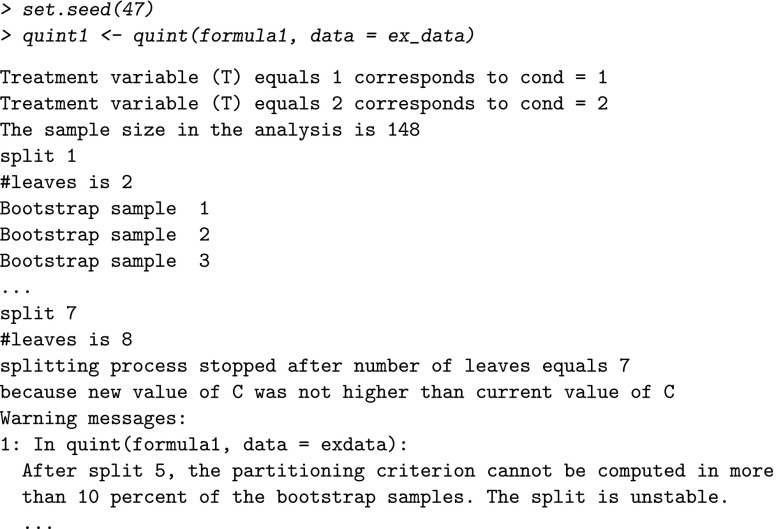


The first two lines of this output explain the relation between the categories of the treatment variable *T* used in the analysis, and the categories of the treatment variable in the data set (in our case variable cond). The third line shows the number of patients that are used in the analysis; these are the patients without missing values on any of the variables included in the formula. Thus, in our example data, 148 out of the total of 168 patients who received nutrition or education therapy have no missing values on the outcome and baseline variables included in formula1. The end of the output shows the reason why the splitting process stopped. In this case, no split 7 could be found that implied a higher value of *C*. For this analysis, the output also gives two warning messages of which one is displayed above. It refers to difficulties in the bootstrap procedure that suggest instability of the tree after split 5. The result of the analysis is an object of class quint, from which the fit, split and leaf information can be obtained using the summary function: 
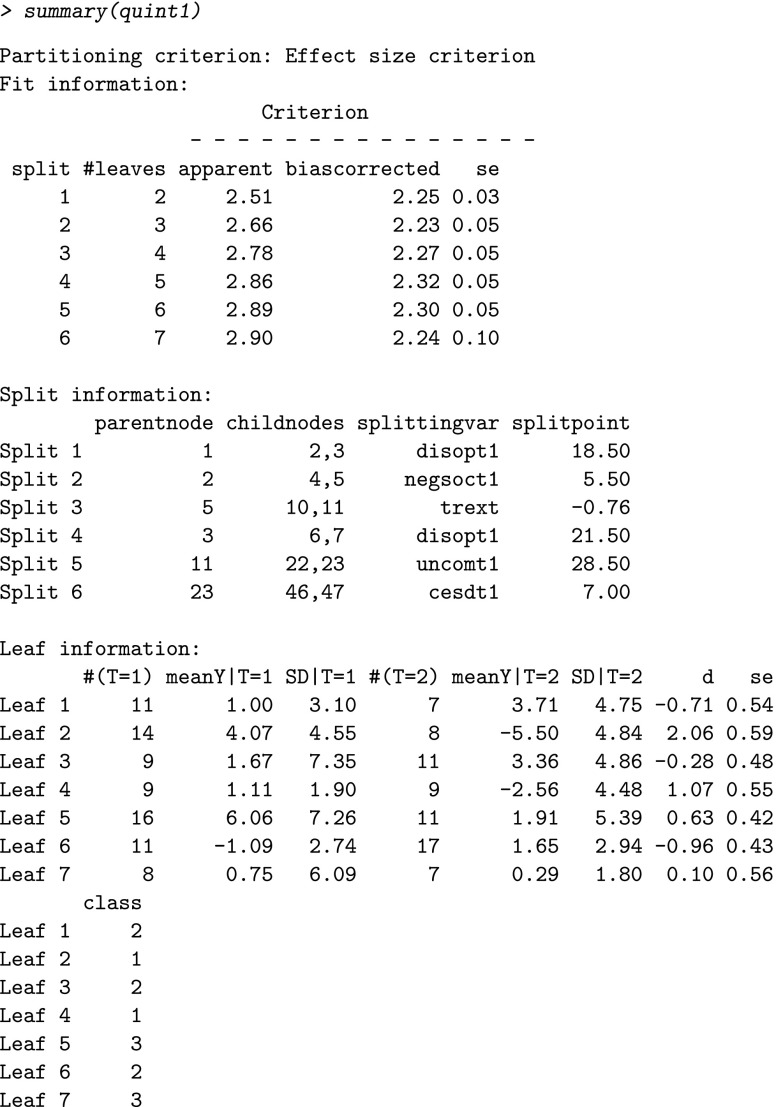


The first line of this output concerns the type of partitioning criterion *C*, in this case the default criterion was used, namely, the Effect size criterion. The fit information of the full tree displays per split the apparent value of *C*, the bias-corrected value of *C* (which resulted from the bootstrap procedure), and the corresponding standard error (*se*). Note that the apparent value of *C* increases with an increasing number of splits (this is always true), whereas the bias-corrected value of *C* reaches its maximum at four splits, and then decreases.

The split information shows in the first two columns the node numbers of the parent nodes that were split and those of the resulting child nodes. The node numbering is the same as the one commonly used (e.g., in Breiman et al., 1984). In the third and fourth column, the splitting variable and corresponding split point are displayed per split.

The leaf information contains standard descriptive statistics (group size, mean outcome, and standard deviation [SD]) for each treatment group per leaf of the full tree (i.e., the tree after six splits). In addition, the effect size *d* (i.e., the standardized mean difference of *T*=1 minus *T*=2), its standard error (se), and the class assignment are displayed. When instead of the Effect size criterion, the Difference in means criterion is used, the same leaf information is given. In this example, the first leaf consists of 11 women from the nutrition condition (*T*=1), with a mean improvement in depression of 1.00 (SD =3.10) and seven women from the education condition (*T*=2), with a mean improvement of 3.71 (SD =4.75). The corresponding effect size *d* equals -0.71 (se =0.54), and the leaf is assigned to *℘*_2_, indicating that for these women education therapy outperforms nutrition therapy.

The fit information (fi), split information (si), and leaf information (li) are stored in three matrices that can also be inspected separately: 
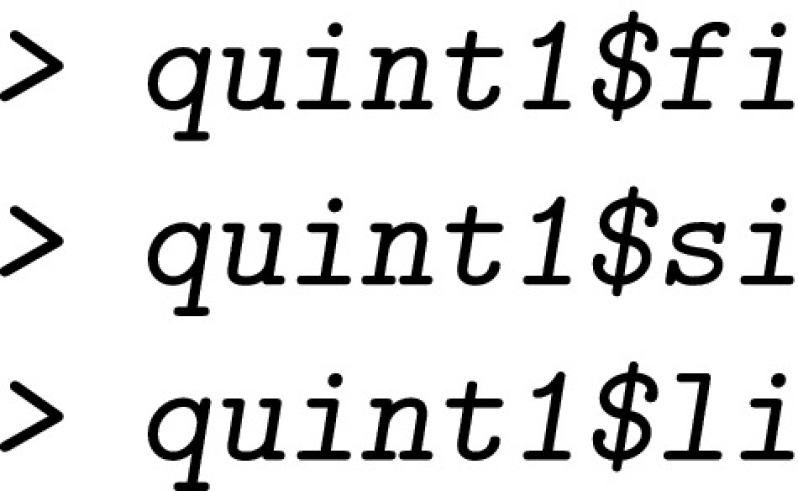


As explained before, the full tree may be too large and needs to be pruned back to avoid overfitting. The best tree is selected automatically by the function prune.quint. The input of this function is the object of the full tree (i.e., quint1). 
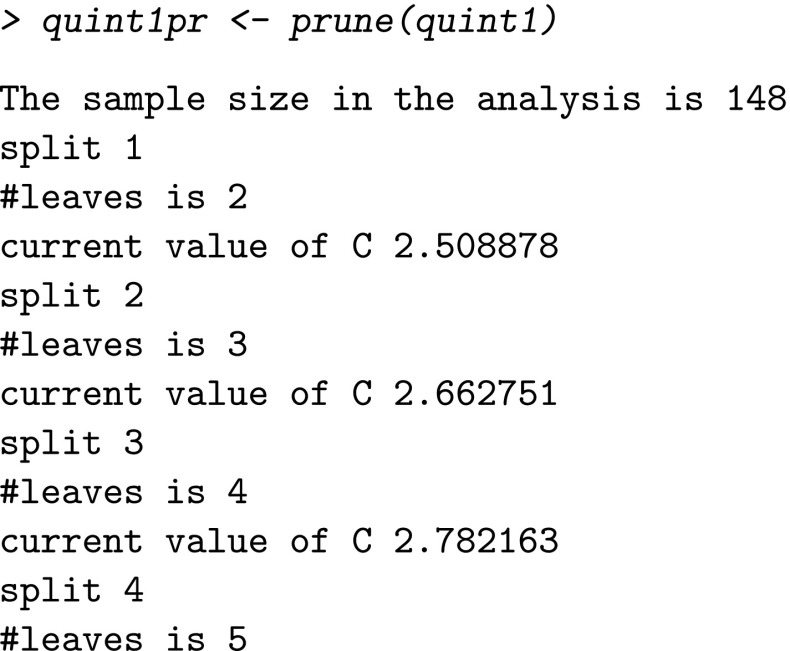


The resulting pruned tree with four splits (i.e., five leaves) is an object of class quint, from which fit, split, and leaf information can be obtained using the summary function, and it can be visualized by plot.quint: 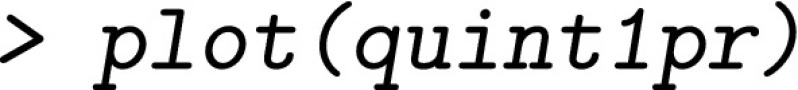


The plot of the pruned tree is displayed in Fig. 1. The inner nodes of the tree contain the labels of the splitting variables, and next to the branches the split points are shown. In the leaves of the tree, the effect sizes *d* are displayed by black dots, along with a 95 *%* confidence interval.

**Fig. 2:**
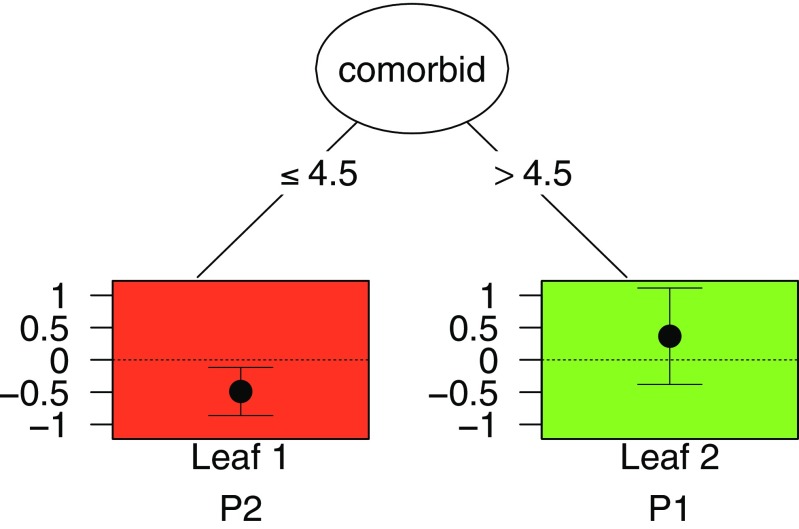
Example of a qualitative interaction tree for the outcome Improvement in physical functioning from the Breast Cancer Recovery Project data, using default values of the tuning parameters. The leaves of the tree are assigned to subgroups *℘*_2_ and *℘*_1_, denoted in the figure by P2 and P1. The *vertical axis* of the leaves pertains to the effect size *d*

For the interpretation of the pruned tree, we inspect the assignment of the leaves to the partition classes and the paths of the tree leading to the leaves. Figure 1 shows that for one group of women (Leaf 2, green) the nutrition intervention outperforms the education intervention, in particular, the nutrition intervention resulted in a higher improvement in depression for those women with a lower level of dispositional optimism, a higher level of negative social interaction, and the least extensive form of primary treatment (i.e., lumpectomy without or with only one form of adjuvant therapy). In contrast, for two groups of women (Leaves 1 and 4, red), the education intervention outperforms the nutrition intervention; one of these groups of women reported a lower level of dispositional optimism and a lower level of negative social interaction, whereas the other group reported a medium level of dispositional optimism. For the remaining types of women (Leaves 3 and 5, grey) both interventions resulted in about the same improvement in depression. To learn more about the exact levels of improvement and the effect sizes, we inspect the leaf information of the pruned tree, rounded at two decimals: 
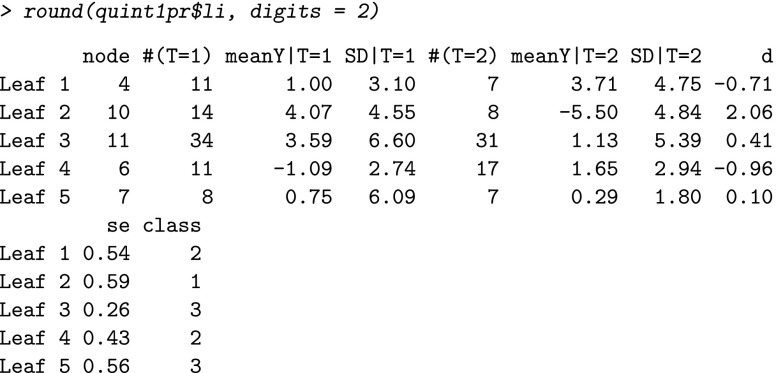


Also, for the second outcome variable, Improvement in physical functioning, a quint analysis with default values of the tuning parameters was performed: 
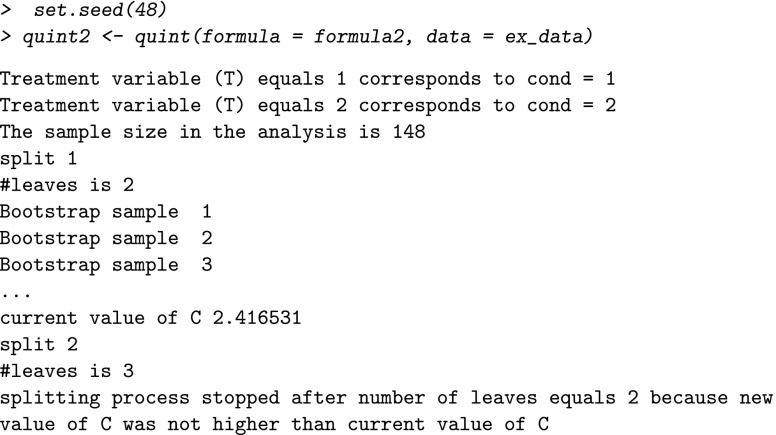


Because the result of this analysis was a tree with just two leaves, there was no need for pruning, and we continued by just inspecting the leaf information and the plot of the tree: 
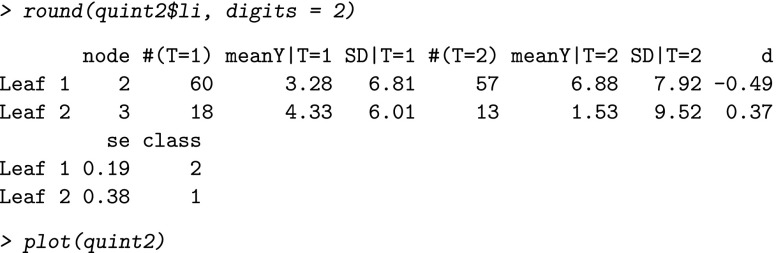


The resulting plot (see Fig. 2) shows that for women with four or fewer comorbidities (Leaf 1, the red one assigned to *℘*_2_) the education intervention was better than the nutrition intervention. The leaf information shows that in this leaf the mean improvement was 3.28 for the nutrition intervention and 6.88 for the education intervention. This latter value was larger, but can be considered as a small improvement from a clinical viewpoint, taking into account the guidelines from Wyrwich et al. (2005).

For women with more than four comorbidities (Leaf 2, the green one assigned to *℘*_1_), the leaf information shows that the nutrition intervention resulted in a larger improvement in physical functioning (i.e., 4.33) than the education intervention (1.53). Yet, 4.33 can also be considered as a small change from a clinical viewpoint.

### Second analyses with modified values for the tuning parameters

Several values of the tuning parameters used in a quint analysis can be adapted by the user. Table 1 gives an overview of all parameters involved, subdivided in those concerning the partitioning criterion, the stopping criterion, the boundary conditions, and the bootstrap procedure. In this section, we will describe how to change the parameters, and the considerations associated with these changes.

**Table 1:** Overview of the tuning parameters that are by quint and can be the user via the function quint.control

Argument	Meaning	Possible values	Default value	Example
	*Partitioning criterion*			
crit	Type of partitioning criterion	“es” (effect size criterion) and “dm”	“es”	crit=“dm”
		(difference in means criterion)		
w	Weights of the Difference in	two positive real numbers, at least	(*w*_1_,*w*_2_)=	w=c(1/log(1 + 2),
	treatment outcome and	one of which should be nonzero	(1/log(1 + 3), 1/log(.50^*^)) or	1/log(.50^*^148))
	Cardinality components		(1/log(IQR(Y)), 1 /log(.50^*^))^*a*^	
	*Stopping criterion*			
maxl	Maximum number of leaves	any integer between 1 and 50	10	maxl = 3
	*Boundary conditions*			
dmin	Minimum absolute value of *d* in each	any real between 0 and 3	0.30	dmin = 0.40
	of the two leaves after the first split			
a1	Minimal sample size of treatment A	any integer between 1 and *n*_1_, where	.10^*^ *n*_1_	a1 = 25
	(*T*=1) in a leaf	*n*_1_ denotes the sample size of *T*=1		
a2	Minimal sample size of treatment B	any integer between 1 and *n*_2_, where	.10^*^ *n*_2_	a2 = 25
	(*T*=2) in a leaf	*n*_1_ denotes the sample size of *T*=2		
	*Bootstrap procedure*			
Bootstrap	Whether to perform bootstrapping	FALSE and TRUE	TRUE	Bootstrap = FALSE
B	Number of bootstrap samples	any integer larger than 1	25	B=50^b^

^a^The default values of the weights are automatically adapted, depending on the choice of the type of partitioning criterion

^b^If the value of *B* is chosen by the user (e.g., *B* = 50), the value of Bootstrap needs to be kept at TRUE

With regard to the partitioning criterion, a first parameter concerns the type of partitioning criterion, that is, the Effect size criterion (which is the default as mentioned before) or the Difference in means criterion. For this choice, one possible consideration concerns the measurement scale of the outcome variable: If the outcome is measured on a scale with values that do not have a well-specified meaning (such as, Improvement in depression), the Effect size criterion may be preferred. In contrast, if a scale is used with values that bear a well-defined clinical interpretation (such as, Improvement in physical functioning), the Difference in means criterion is to be preferred. Another consideration pertains to whether and how one is willing to take into account subgroup heterogeneity. If one wants to identify subgroups that are homogeneous in treatment effect, then the Effect size criterion is to be preferred (note that an effect size of the same difference in means is larger when the pooled standard deviation of the treatment groups is smaller); if, in contrast, the only research concern is to identify subgroups with a mean difference in treatment outcome that is as large as possible, then the Difference in means criterion is to be preferred. A final consideration pertains to the robustness of the results. Baguley (2009) showed that the raw difference in means is more robust than the standardized effect size.

A second parameter concerns the weights of the two components of the partitioning criterion, the Difference in treatment outcome and the Cardinality component, that is, *w*_1_ and *w*_2_ (see also formula 6 in Dusseldorp & Van Mechelen, 2014). As mentioned before (see section Goal of QUINT), the Cardinality component concerns the sample sizes of the leaves assigned to *℘*_1_ and *℘*_2_. The default weights are chosen in such a way that the two components are weighted equally with the maximum possible value for each component being 2. The default value of *w*_1_ depends on the criterion that is used: if this is the Difference in means criterion, the default value of *w*_1_ is put equal to 1/log(*IQR*(*Y*)), where IQR denotes the interquartile range (which can be considered as a plausible maximum value for the difference in means). If the Effect size criterion is used, the default value of *w*_1_ is put equal to 1/log(1+3), with 3 being considered as a plausible maximum value of the effect size. In a specific research field this value may be typically lower (e.g., 2), and the weight can be changed accordingly (see Table 1 for an example).

We change the values of the tuning parameters, using the function quint.control. For example, if we want to use the Difference in means criterion for Improvement in physical functioning, we first make a new control object, and then we use this control object in the analysis: 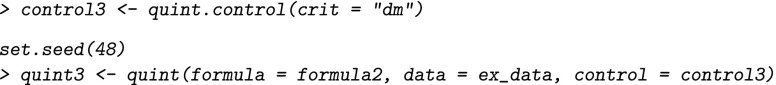


For this example, the resulting tree is the same as the tree grown with the Effect size criterion. In our experience this is often the case, but subtle differences may occur.

With regard to the stopping criterion, the maximum number of leaves of the tree can be changed. This enables the user to stop the tree algorithm before the maximum value of the partitioning criterion *C* was reached, for example, to inspect a tree of a certain fixed size (e.g., two leaves). The default value is set at ten leaves (which most of the times suffices in practice because the maximum value of *C* is reached earlier). This value can be changed into, for example, two leaves using the following commands: 
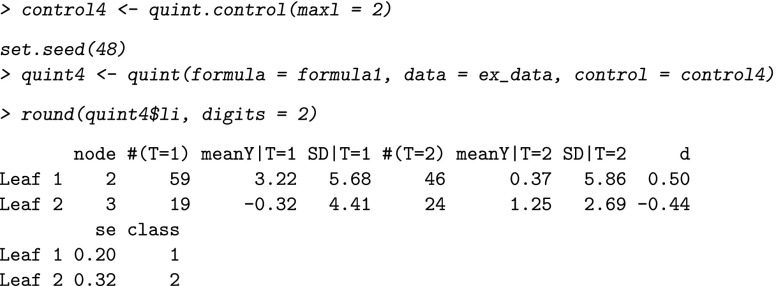


With regard to the boundary conditions, a first tuning parameter concerns the critical minimum value of the absolute effect size in each leaf (*d*_*min*_) that is checked by the algorithm after the first split (i.e., the qualitative interaction condition). The results of an extensive simulation study (Dusseldorp & Van Mechelen, 2014) showed that a good balance between type I error and type II error is obtained for *d*_*min*_=0.30 and *N*=400. Therefore, the default value of *d*_*min*_ equals 0.30. For smaller sample sizes, a higher value of *d*_*min*_ is recommended to control for the risk of finding spurious interactions. In our example with a sample size of *N*=148, it may be advisable to increase the value to 0.40. For Improvement in depression, this change will not influence the result, because the effect sizes in the two leaves after the first split (see output above) are both greater than 0.40. However, if we change *d*_*min*_ to 0.40 for Improvement in physical functioning, we obtain the following result: 
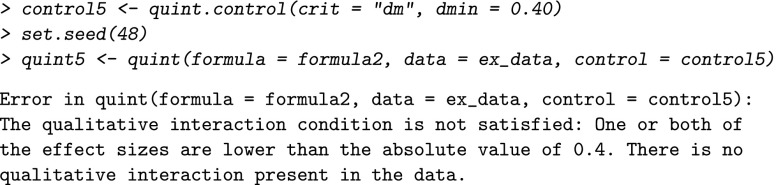


The error message shows that the qualitative interaction condition (as explained at length in the section on the QUINT algorithm) is violated, and, as a consequence, no tree is grown. This result suggests that the interaction we found earlier for Improvement in physical functioning using the default values, may be a spurious one.

The remaining tuning parameters associated with the boundary conditions concern the minimal sample size per treatment condition in *T*=1 (a1), and in *T*=2 (a2). The default values have been set at 10 *%* of the treatment group sample sizes. However, the user is free to choose any value as minimum treatment sample size. When on the one hand treatment sample sizes are relatively small, 10 *%* of them may not allow to estimate the mean outcome in a treatment group with sufficient confidence. When on the other hand treatment sample sizes are large (i.e., 500 or more), we recommend to choose a lower value than the default to avoid that the tree algorithm stops (too) early (see Table 1 for an example).

With regard to the bootstrap procedure, a first tuning parameter determines whether or not this procedure is performed. If bootstrapping is not performed, the computation time of quint is much shorter, yet at the expense of a lack of information on the amount of overfitting. A second tuning parameter concerns the number of bootstrap samples, with a higher number (e.g., *B*=200) leading to more stable results. The default value has been put to *B*=25 (i.e., the recommended minimum value by LeBlanc & Crowley, 1993).

### Discussion

We proposed a new R package quint that can be used to study the important clinical problem of differential treatment efficacy. When many client characteristics (or other baseline characteristics) have been measured that may moderate treatment outcome, the problem of subgroup identification is a very difficult one with a high risk of type I and type II errors. In such a situation, the package quint can be most useful through its versatile way of searching for subgroups and its procedures that control for inferential errors and overfitting. The quint analysis focuses especially on the identification of subgroups that are involved in so-called qualitative treatment subgroup interactions. This type of interactions implies that for some subgroup of clients, one treatment alternative outperforms another while for another subgroup the reverse holds, and is therefore of utmost importance for personalized treatment assignment. It should be noted that a quint analysis does not aim at identifying quantitative interactions. If data contain no qualitative interactions, no tree will be grown by quint. In this paper, we demonstrated the functions of the package using data from the Breast Cancer Recovery Project, and highlighted possibilities to direct the analysis on the basis of theoretical and practical considerations.

The R package quint can be used for data from a randomized controlled trial. In this paper, we focused on a clinical trial involving cancer patients, but the method is applicable to controlled experiments in any setting, such as randomized experiments in which two interventions, training programs, or any other type of experimental manipulations are compared (e.g., Taylor, Davis, & Maxwell, 2001), including controlled web-based experiments (so-called A/B tests) in marketing research (Kohavi, Longbotham, Sommerfield, & Henne, 2009). Most important features of the data are that the persons are randomly assigned to two conditions (A and B) and that the person characteristics are measured before the treatment is received (unless it is very unlikely that the treatment has altered the characteristic, e.g., gender or age in years). Also, a total sample size of higher or equal to 400 is recommended, based on results from a simulation study (Dusseldorp and Van Mechelen 2014), to allow for the study of more complex treatment-subgroup interactions.

The core idea of random assignment of clients to treatment groups, is that the clients only differ with respect to the treatment variable. This implies that the client characteristics are not associated with the treatment variable and it enables that the observed differences in the (sub)groups can be attributed to the differences in treatment. However, this does not imply that the result of a subgroup analysis, such as the tree found by quint, is always generalizable towards the full client population. In some cases, indeed, the distribution of some characteristics in the study sample may not be the same as those in the population. For example, our sample might consist for 20 *%* of male clients, while the population to which we want to generalize consists for 50 *%* of male clients. One possible solution to take this imbalance into account, is to incorporate weighting in the analysis by quint. A vector of weights can easily be implemented for the Difference in means criterion of quint. For the Effect size criterion, this is more difficult, due to the estimation of a pooled standard deviation.

The current implementation of quint has several limitations: (a) weighting of clients according to some known population distribution is not possible; (b) clients with one or more missing values on any of the variables are omitted from the analysis (so-called listwise deletion); (c) the outcome variable should be numeric, and (d) categorical baseline characteristics involving more than two categories cannot be handled by the software. Currently, we are working on a new version of the R package that can deal with categorical baseline characteristics involving more than two categories.

Because QUINT is a post hoc method, it is recommended for clinical practice to check whether the results of QUINT can be replicated in a new randomized controlled trial. Ideally, for the sampling of the participants in this new trial a stratified sampling scheme should be used with stratification on the patterns of moderator variables identified by QUINT.

## Figures and Tables

**Fig. 3:**
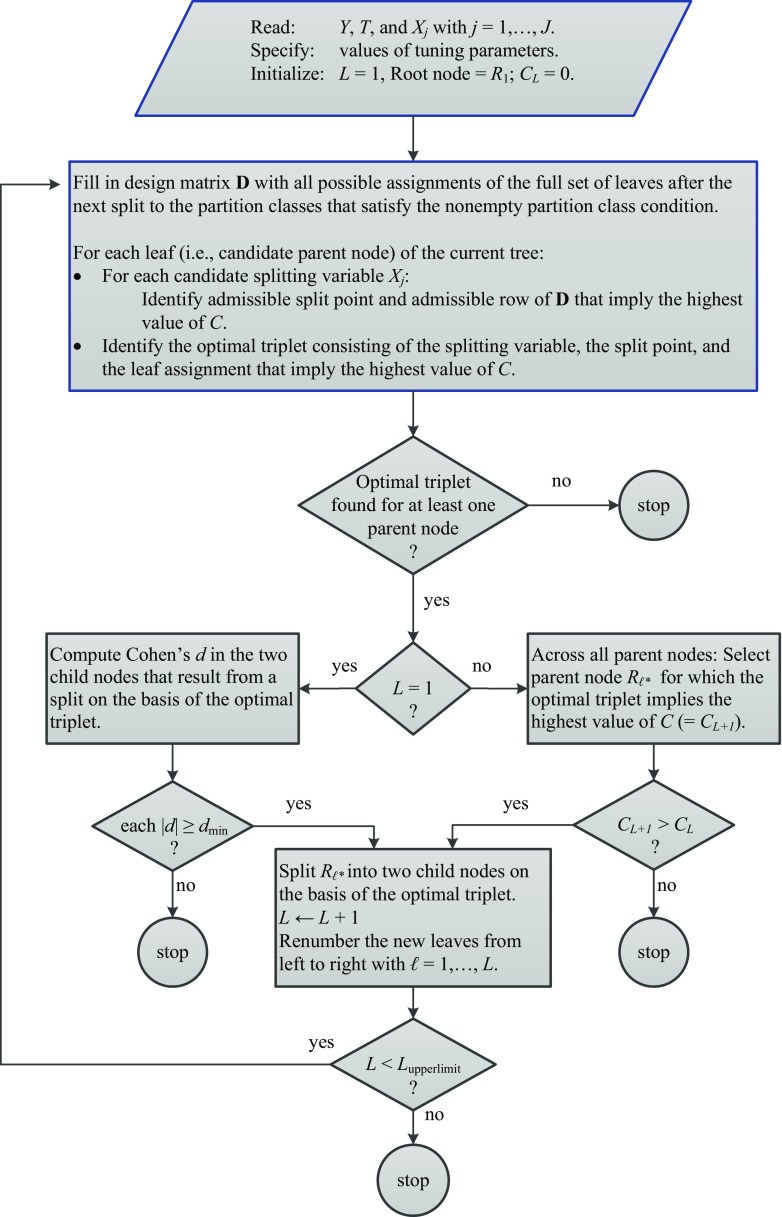
Flowchart of the algorithm for tree growing underlying quint

## Appendix

In this Appendix, a detailed description of the algorithm underlying the quint function is given using a flowchart (see Fig. 3). In a preliminary step, the data are read, several tuning parameters need to be specified (with the default values being given in Table 1), and three variables are initialized, that is: the total current number of leaves *L* is put equal to 1, the root node is given number 1, and the current value of the QUINT criterion *C* is put equal to 0. The algorithm then continues with the actual splitting procedure. As a preparatory step in this procedure, a design matrix **D** of size (3^*L*+1^-2^*L*+2^+1)×(*L*+1) is constructed, each row of which contains a theoretically possible assignment of all leaves that will result after the next split to the three partition classes; at this point “theoretically possible” simply means that both *℘*_1_ and *℘*_2_ are nonempty (which implies that at least one leaf should be assigned to each of the two classes in question).

After this preparatory step, the splitting procedure continues as follows: Each leaf of the current tree is considered as a candidate parent node for the next split. Given a candidate parent node, the algorithm looks for the best possible split of that node in terms of an optimal combination of three ingredients (i.e., a so-called optimal triplet): a splitting variable *X*_*j*_, an admissible split point, and an admissible assignment (i.e., a row of **D**), with a split point and an assignment being admissible if: 1) each of the two leaves resulting from the split contains a pre-specified minimum number of clients assigned to treatment A and B, and 2) any leaf can only be assigned to *℘*_1_ if in that leaf the mean outcome of the clients in treatment A is higher than the mean outcome of those in treatment B (and vice versa for *℘*_2_); the optimal triplet then is the one that implies the highest value of the criterion *C*.

Next, if *L*=1 then the flowchart shows that the qualitative interaction condition (i.e., one of the boundary conditions) is to be checked. If this condition is satisfied, the root node is split, the new value of *L* is set at 2, the new set of leaves is renumbered, and the splitting procedure is repeated. If *L*>1 then the values of *C* are compared across all candidate parent nodes, and the node with the highest value is chosen as the node to be split ($R_{\ell ^{\ast }}$). If the value of *C* implied by the split of ($R_{\ell ^{\ast }}$) exceeds the current criterion value, then ($R_{\ell ^{\ast }}$) is split effectively, the new value of *L* is increased by one unit, the new set of leaves is renumbered, and the splitting procedure is repeated.

The tree growing stops: (1) if no candidate parent node can be found with an admissible triplet (upper circle on the right side of the flowchart), (2) if the qualitative interaction criterion is not met (lower left circle in the flowchart), (3) if the value of *C* implied by the split of $R_{\ell ^{\ast }}$ does not exceed the current value of *C* (i.e., lower right circle in the flowchart), or (4) if the current total number of leaves equals a user-specified maximum, *L*_*u**p**p**e**r**l**i**m**i**t*_ (i.e., circle at the end of the flowchart). (Note that *L*_*u**p**p**e**r**l**i**m**i**t*_ is called maxl in the R package quint.)

## References

[CR1] Baguley T (2009). Standardized or simple effect size: What should be reported?. British Journal of Psychology.

[CR2] Breiman L, Friedman J, Olshen R, Stone C (1984). Classification and regression trees..

[CR3] Byar D (1985). Assessing apparent treatment-covariate interactions in randomized clinical trials. Statistics in Medicine.

[CR4] Cohen J (1988). Statistical power analysis for the behavioral sciences.

[CR5] Cohen J, Cohen P, West S, Aiken L (2003). Applied multiple regression/correlation analysis for the behavioral sciences..

[CR6] Doove L, Dusseldorp E, Van Deun K, Van Mechelen I (2014). A comparison of five recursive partitioning methods to find person subgroups involved in meaningful treatment–subgroup interactions.. Advances in Data Analysis and Classification.

[CR7] Dusseldorp E, Conversano C, Van Os BJ (2010). Combining an additive and tree-based regression model simultaneously: Stima.. Journal of Computational and Graphical Statistics.

[CR8] Dusseldorp E, Meulman JJ (2004). The regression trunk approach to discover treatment covariate interaction.. Psychometrika.

[CR9] Dusseldorp E, Van Mechelen I (2014). Qualitative interaction trees: A tool to identify qualitative treatment-subgroup interactions.. Statistics in Medicine.

[CR10] Fierz W (2004). Challenge of personalized health care: To what extent is medicine already individualized and what are the future trends?. Medical Science Monitor.

[CR11] Foster J, Taylor J, Ruberg S (2011). Subgroup identification from randomized clinical trial data.. Statistics in Medicine.

[CR12] Fritz HL, Helgeson VS (1998). Distinctions of unmitigated communion from communion: self-neglect and over-involvement with others. Journal of Personality and Social Psychology.

[CR13] Hastie TJ, Tibshirani RJ, Friedman JH (2001). The elements of statistical learning..

[CR14] Kohavi R, Longbotham R, Sommerfield D, Henne RM (2009). Controlled experiments on the web: survey and practical guide.. Data mining and knowledge discovery.

[CR15] Kraemer H, Wilson G, Fairbun C, Agras W (2002). Mediators and moderators of treatment effects in randomized clinical trials.. Archives of General Psychiatry.

[CR16] LeBlanc M, Crowley J (1993). Survival trees by goodness of split.. Journal of the American Statistical Association.

[CR17] Lipkovich I, Dmitrienko A, Denne J, Enas G (2011). Subgroup identification based on differential effect search–a recursive partitioning method for establishing response to treatment in patient subpopulations.. Statistics in Medicine.

[CR18] Lubin A (1961). The interpretation of significant interaction.. Educational and Psychological Measurement.

[CR19] R Core Team (2014). R: A language and environment for statistical computing [Manuel de logiciel]. Vienna, Austria. Retrieved from http://www.R-project.org/

[CR20] Radloff LS (1977). The CES-D scale a self-report depression scale for research in the general population.. Applied Psychological Measurement.

[CR21] Roth A, Fonagy P (2006). What works for whom?: A critical review of psychotherapy research..

[CR22] Scheier MF, Carver CS, Bridges MW (1994). Distinguishing optimism from neuroticism (and trait anxiety, self-mastery, and self-esteem): a reevaluation of the life orientation test.. Journal of Personality and Social Psychology.

[CR23] Scheier, M. F., Helgeson, V. S., Schulz, R., Colvin, S., Berga, S., Bridges, M. W., Knapp, J., Gerszten, K., & Pappert, W. S. (2005). Interventions to enhance physical and psychological functioning among younger women who are ending nonhormonal adjuvant treatment for early stage breast cancer. *Journal of Clinical Oncology, 23*(19), 4298–4311. 10.1200/JCO.2005.05.362 15994143

[CR24] Scheier MF, Helgeson VS, Schulz R, Colvin S, Berga SL, Knapp J, Gerszten K (2007). Moderators of interventions designed to enhance physical and psychological functioning among younger women with early stage breast cancer.. Journal of Clinical Oncology.

[CR25] Shaffer J (1991). Probability of directional errors with disordinal (qualitative) interaction.. Psychometrika.

[CR26] Shuster J, van Eys J (1983). Interaction between prognostic factors and treatment. Controlled Clinical Trials.

[CR27] Su X, Tsai CL, Wang H, Nickerson DM, Li B (2009). Subgroup analysis via recursive partitioning.. The Journal of Machine Learning Research.

[CR28] Taylor BG, Davis RC, Maxwell CD (2001). The effects of a group batterer treatment program: a randomized experiment in Brooklyn.. Justice Quarterly.

[CR29] Ware Jr, J. E., & Sherbourne, C. D. (1992). The MOS 36-item shortform health survey (SF-36): I. conceptual framework and item selection. *Medical Care, 30*, 473–483. 1593914

[CR30] Wyrwich KW, Bullinger M, Aaronson N, Hays RD, Patrick DL, Symonds T (2005). Estimating clinically significant differences in quality of life outcomes.. Quality of Life Research.

[CR31] Zeileis A, Hothorn T, Hornik K (2008). Model-based recursive partitioning.. Journal of Computational and Graphical Statistics.

